# The Association Between Genetics and Response to Treatment with Biologics in Patients with Psoriasis

**DOI:** 10.3390/ijms26188998

**Published:** 2025-09-16

**Authors:** Nikolai Loft, Sule Altintas, Rownaq Al-Sofi, Daniel Isufi, Claus Zachariae, Diljit Kaur-Knudsen, Claus Henrik Nielsen, Lone Skov

**Affiliations:** 1 Department of Dermatology and Allergy, Copenhagen University Hospital-Herlev and Gentofte, 2900 Hellerup, Denmark; sule.altintas@regionh.dk (S.A.); rownaq96@hotmail.com (R.A.-S.); daniel.isufi@regionh.dk (D.I.); claus.zachariae@regionh.dk (C.Z.); diljit.kaur.knudsen.02@regionh.dk (D.K.-K.); lone.skov.02@regionh.dk (L.S.); 2 Copenhagen Research Group for Inflammatory Skin, Herlev and Gentofte Hospital, 2900 Hellerup, Denmark; 3 Department of Clinical Medicine, Faculty of Health and Medical Sciences, University of Copenhagen, 1172 Copenhagen, Denmark; 4 Institute for Inflammation Research, Center for Rheumatology and Spine Diseases, Copenhagen University Hospital, Rigshospitalet, 2100 Copenhagen, Denmark; claushnielsen@hotmail.com

**Keywords:** psoriasis, biologics, pharmacogenetics, genetics, adalimumab, ustekinumab, secukinumab, ixekizumab, brodalumab

## Abstract

Biologics targeting tumor necrosis factor-α (TNFi), interleukin-12/23 (IL-12/23i), and interleukin-17 cytokine or receptor (IL-17i/IL-17Ri) have transformed psoriasis management. However, interindividual variation in response underscores the need for predictive biomarkers in guiding therapy selection. Patients treated with a biologic for psoriasis were genotyped for 67 single-nucleotide polymorphisms (SNPs) previously associated with response to biologics. Odds ratios (OR) with 95% confidence intervals (CI) for associations between SNPs and response to biologics were calculated using logistic regression models with an absolute Psoriasis Area and Severity Index (PASI) ≤2 after 3 months as treatment response. A *p*-value < 0.05 was considered statistically significant. In total, 373 patients with 574 treatment series were included. Twelve SNPs were associated with treatment response: four uniquely with response to TNF inhibitors (TNFi), two to IL-12/23i, and five to IL-17i/IL-17Ri, while one was associated with response to both TNFi and IL-17i/IL-17Ri. Notably, IRAK3 (rs11541076) and CD84 (rs6427528) were associated with response to TNFi (OR: 2.56 [95% CI: 1.22–5.37], *p* = 0.012 and OR: 0.53 [95% CI: 0.30–0.91], *p* = 0.023) and IL-17i/IL-17Ri (OR: 2.55 [95% CI: 0.70–9.22], *p* = 0.15), and OR: 0.50 [95% CI: 0.25–0.98], *p* = 0.045), with trends toward opposite associations for IL-12/23i (OR: 0.38 [95%CI: 0.08–1.72], *p* = 0.21 and OR: 1.64 [95%CI: 0.68–3.93], *p* = 0.26). This study replicates known genetic associations with biologic response in psoriasis. Variants in IRAK3 and CD84 show potential as stratification biomarkers, although they need confirmation in independent cohorts.

## 1. Introduction

Treatment of psoriasis has been revolutionized with the introduction of biologics and today several biologics, target specific cytokines in the psoriasis pathogenesis, including drugs targeting tumor necrosis factor alpha (TNF-α), interleukin (IL)-12p40, IL-17, and IL-23p19 [1]. Initiation of these treatments are often according to national guidelines considering severity of disease, price, and comorbidities including psoriatic arthritis (PsA) and inflammatory bowel disease (IBD) [2,3]. While most patients respond to therapy, a considerable proportion of patients do not and thereby require a switch of treatment [4]. Patients with previous exposure to biologics often have a lower chance of response to the subsequent biologic [4,5] and, in contrast, patients with early good response have a lower risk of flares and drug discontinuation [6]. This highlights the need for identifying biomarkers that can be used for identifying patients with the highest chance of responding to a specific treatment. Genetic biomarkers have been proposed as a way of stratifying patients to treatment [7]. Indeed, the human leukocyte antigen (HLA)-C:06:02 genotype has consecutively been associated with good response to treatment with IL-12/23 inhibitors, while it does not appear to be associated with response to other biologics [8,9,10,11]. Other genetic variants have sparsely been investigated in smaller cohorts, and the findings have seldomly been replicated [12]. Therefore, we aimed to assess and replicate the association with the response of genetic variants outside the HLA region that have previously been associated with response to biologics in psoriasis and other chronic inflammatory diseases.

## 2. Results

In total, 373 patients with 574 treatment series were included (Table 1). Patients had a treatment series with TNFi (*n* = 319), IL-12/23i (*n* = 109), or an IL-17i (*n* = 146). Mean age was 45.3 (SD 15.1), 256 were male (69%), mean BMI was 28.9 (SD 6.4), mean baseline PASI 9.5 (SD 5.5), and 76 (20%) had concomitant PsA.

### 2.1. Association Between Genetics Variants and Response to TNFi

**Figure 1:**
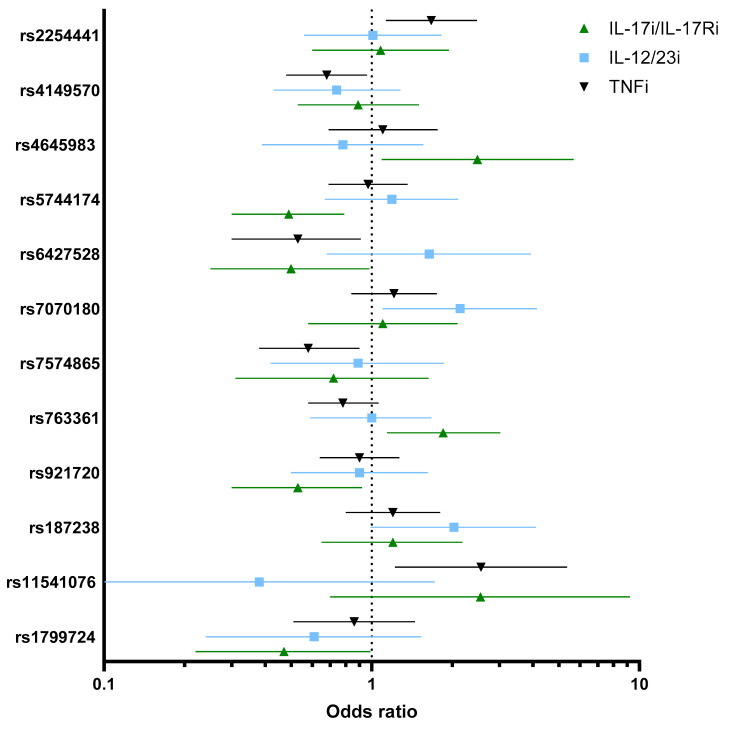
Odds ratios with 95% confidence interval for statistically significant associations between genetic variants and response to treatment after 3 months with tumor necrosis factor α inhibitors (TNFi), interleukin (IL)-12/23i, and IL-17i/IL-17Ri.

In total, 319 patients were treated with TNFi, of whom 130 (40.8%) achieved an absolute PASI ≤ 2 after 3 months of treatment. Most patients were biologic-naïve (228, 72%) and treated with adalimumab (269, 84%). Of the SNPs examined, five were associated with response or non-response to treatment with TNFi (Supplementary Table S1). The minor alleles of IRAK3 (*rs11541076A/T*) (OR: 2.56 [95% CI: 1.22–5.37], *p* = 0.012) and PSTPIP1 (*rs2254441G/A*) (OR: 1.67 [95% CI: 1.13–2.47], *p* = 0.0096) were associated with good response to TNFi (Table 2, Figure 1). The minor alleles of TNFRSF1A (*rs4149570 C/A*) (OR: 0.68 [95% CI: 0.48–0.96], *p* = 0.032), CD84 (*rs6427528G/A*) (0.53 [95% CI: 0.30–0.91], *p* = 0.023), and STAT4 (*rs7574865 G/T*) (OR: 0.58 [95% CI: 0.38–0.90], *p* = 0.014) were associated with non-response to TNFi. Adjustment of the results for sex, age, psoriatic arthritis, and biologic nativity was included.

### 2.2. Association Between Genetic Variants and Response to IL-12/23i

In total, 109 patients were treated with IL-12/23i, all of whom were treated with ustekinumab, and 49 (45.0%) achieved an absolute PASI ≤ 2 after 3 months of treatment. Of the treated patients, 59 (55.0%) were biologic-naïve. Of the examined SNPs, two were associated with response to treatment with IL-12/23i. The minor allele of GFRA1 (*rs7070180 C/T*) was associated with good response to IL-12/23i (OR: 2.14 [95% CI: 1.10–4.14], *p* = 0.024) both in crude and adjusted analysis, and the minor allele of IL-18 (*rs187238C/G*) was associated with good response to IL-12/23i after adjusting for sex, age, PsA, and biologic naivety (OR: 2.59 (95% CI: 1.11–6.02), *p* = 0.026). None of the SNPs associated with response to TNFi were significantly associated with response to IL-12/23i (Table 2, Figure 1). However, for the minor allele of IRAK3 (*rs11541076A*/T), which was associated with good response to TNFi, a non-statistically significant association with non-response to IL-12/23i was observed (OR: 0.38 [95% CI: 0.08–1.72], *p* = 0.21). Moreover, CD84 (*rs6427528G*/A), which was associated with non-response to TNFi, showed a non-statistically significant association with good response to IL-12/23i (OR: 1.64 [95% CI: 0.68–3.93], *p* = 0.26).

### 2.3. Association Between Genetic Variants and Response to IL-17i/IL-17Ri

In total, 146 patients were treated with IL-17i/IL-17Ri, of whom 99 (68%) achieved an absolute PASI ≤ 2 after 3 months of treatment. Patients were treated with either secukinumab (61, 42%), ixekizumab (55, 38%), or brodalumab (29, 20%). Most patients were biologic experienced, with only 39 patients (26.7%) being biologic-naïve. Of the SNPs examined, six were associated with response or non-response to treatment with IL-17i/IL-17Ri. The minor alleles of CASP9 (*rs4645983G/A*) (OR: 2.48 [95% CI: 1.09–5.68], *p* = 0.030), TLR5 (*rs5744174A/G*) (OR: 1.67 [95% CI: 1.13–2.47], *p* = 0.0096), and CD226 (*rs763361C/T*) (OR: 1.82 [95% CI: 1.14–3.02], *p* = 0.012) were associated with good response to IL-17i/IL-17Ri. The minor alleles of CD84 (*rs6427528G/A*) (0.50 [95% CI: 0.25–0.98], *p* = 0.045), LINC02964 (*rs921720G/A*) (OR: 0.53 [95% CI: 0.30–0.92], *p* = 0.025), and TNF-857 (*rs1799724C/T*) (OR: 0.47 [95% CI: 0.22–0.99], *p* = 0.049) were associated with non-response to IL-17i. None of the SNPs associated with response to IL-12/23i were associated with response to IL-17i. The minor allele of IRAK3 (*rs11541076 A/T*) was, albeit not statistically significant, associated with good response (OR: 2.55 [95% CI:0.70–9.22], *p* = 0.15) to IL-17i/IL-17Ri, as it was with good response to TNFi (Table 2, Figure 1).

## 3. Discussion

In this cohort study of 373 patients treated with 574 series of biologics, we found that 12 SNPs were associated with response to biologics: four uniquely associated with response to TNFi, two with response to IL-12/-23i, five with response to IL-17i/IL-17Ri, while one was associated with response to both TNFi and IL-17i/IL-17Ri. The observed associations were found in genes related to toll like receptor (TLR) and TNF signaling, transmembrane immunoglobulin superfamily receptors, apoptosis regulation, signaling via STAT 3/4, as well as in other genes related to regulation of the innate immune system.

Most previous studies on genetic variants outside HLA associated with response to biologics have been on TNFi, and only a few have addressed response to IL-12/-23i and IL-17i [12]. For a genetic variant to be of clinical use, it needs to fulfill certain criteria, including consistency in association and the ability to stratify treatments, i.e., patients having the genetic variant are more likely to respond to one therapy than to another. The aim of the study was to validate previously reported associations between genetic variants and treatment response to biologics and to investigate their potential association with response to IL-17i or IL-12/-23i. Of the 12 SNPs associated with response to biologics, the genetic variants in IRAK-3 and in CD84 are of particular interest. IRAK3 encodes IL-1 receptor-associated kinase-3 (IRAK-3/IRAK-M), a negative regulator of TLR signaling that prevents the dissociation of IRAK-1 and IRAK-4 from MyD88, thereby hindering further signaling and activation of transcription factors (NF-κB and the MAP kinases) [13]. Carriage of the minor allele of the genetic variant IRAK-3 (*rs11541076*) has previously been associated with response to TNFi in two studies totaling 1916 patients with rheumatoid arthritis [14,15] and a study on 118 patients with ankylosing spondylitis or PsA [16]. However, a study with 376 patients with psoriasis did not show an association with response to TNFi but, on the other hand, showed an association with non-response to ustekinumab among 230 patients with psoriasis [17]. In line with this, we found a trend towards an association with non-response for ustekinumab. Although the functionality of the genetic variant is unknown, TNFis have been shown to reduce IRAK3 expression in monocytes from patients with acute coronary syndrome in vitro [18].

CD84 is a single-pass transmembrane protein that contains an extracellular domain with an immunoglobulin-like (Ig-like) structure. It is part of the SLAM (signaling lymphocytic activation molecule) family, which includes other molecules like SLAMF1, SLAMF3, and SLAMF7, and it is primarily expressed on the surface of T cells, B cells, macrophages, dendritic cells, and natural killer (NK) cells [19]. A minor allele in the CD84 gene (*rs6427528*) has previously been associated with response to etanercept with genome-wide significance in a study including 2706 patients with rheumatoid arthritis in which no association was observed for adalimumab or infliximab [20]. Likewise, a study of 234 patients with psoriasis found the variant to be associated with better response to etanercept but did not show an association with response for adalimumab or ustekinumab [21]. This is in contrast to a study of 132 patients in which the minor allele was associated with longer drug retention for ustekinumab but not for TNFi [22]. Two other studies with 788 and 581 patients with rheumatoid arthritis treated with TNFi could not replicate previous findings of an association with a minor allele in CD84 (*rs6427528*) and response to TNFi [23,24]. In the current study, most patients were treated with adalimumab, and the inconsistency across previous studies warrants cautiousness. Interestingly, the current study is the first to suggest CD84 (*rs6427528*) to be associated with response to IL-17i/IL-17Ri. We also observed a trend toward an association between this SNP and non-response to ustekinumab, suggesting its potential relevance for treatment stratification. Nevertheless, further studies are needed to confirm these findings.

Although most genetic variants were associated with response to only one therapy, these findings may still be relevant. Given the typically small effect sizes of individual variants and the large number of analyses conducted in genetic studies, previously reported associations may have occurred by chance. Therefore, replication in independent cohorts is essential to validate these results. In our study, the strongest of the associations was observed for PSTP1p1 (*rs2254441*), which has previously been found to be associated with response to TNFi in a meta-analysis of patients with psoriasis [12,25,26], supporting the notion of an association with response. Another genetic variant with a plausible association with response is a minor allele in TNFRSF1A (*rs4149570*), which, as in our study, has been associated with poor response to TNFi in two other studies [12]. A minor allele in TNF-857 (*rs1799724*) has been found to be associated with non-response in treatment with TNFi in a meta-analysis [12], but although the direction of the association was similar to that of our study, it was not statistically significant in the current study. Interestingly, in the current study we found this genetic variant to be associated with non-response to IL-17i/IL-17Ri, which could indicate this variant being used for treatment stratification. Additionally, we found several genetic variants for IL-17i/IL-17Ri, which have not previously been associated with response to these treatments. A minor allele in TLR5 (*rs5744174*) has previously been associated with response to ustekinumab in psoriasis [7] and TNFi in rheumatoid arthritis [15,27] and inflammatory bowel diseases [28]. Interestingly, we found no association between TLR5 (*rs5744174*) and response to TNFi or ustekinumab, but an association was observed with IL-17i/IL-17Ri, complicating interpretations. However, the recurrent association between TLR5 (*rs5744174*) and treatment response suggests a potential link between TLR signaling and response to biologics, warranting further investigation. Noteworthy, a large study including 5218 patients across several indications found no study-wide-significant (*p* < 1.25 × 10^−8^) alleles associated with response to IL-17i [29]. However, the study was most likely underpowered to detect the small effect sizes of individual SNPs in these heterogenous populations spanning several chronic immune-mediated diseases and the extremely stringent significance threshold exceeding that of a standard genome-wide significance. Nevertheless, these findings underscore the importance of conducting multiple, well-powered studies aimed at replicating previous associations.

This study is prone to several limitations. Firstly, we did not have the power to detect small effect sizes, and as none of the found associations would withstand Bonferroni corrections, we cannot refute some of the found associations to be false positives and be due to chance. However, as the aim of the study was to assess and confirm previous associations, we reported uncorrected *p*-values. Still, the observed associations need further validation before conclusions can be drawn. For power reasons, associations were assessed according to treatment class. It is important to note that the genetic associations we observed may not be entirely representative of the drug class but could instead reflect drug-specific effects. Additionally, patients treated with IL-17i/IL-17Ri were in most cases biologically exposed, thus representing a group with more difficult-to-treat psoriasis, which might have influenced both clinical response and the detection of genetic associations. Although little is known regarding the genetics of difficult-to-treat psoriasis [30], the generalizability of our findings might be limited to biologic-naïve patients. Our study was not powered to perform a robust subgroup analysis stratified by biologic exposure, and this remains an important factor for future research. A limitation of our study is that the cohort was recruited from a single hospital in Denmark, and the majority of patients were of European ancestry. This may restrict the generalizability of our findings to other populations, as allele frequencies and genetic architectures can vary across ancestries. Furthermore, some data were missing, but this was most likely missing at random. Strengths of the study include the relatively large number of patients, evaluation of multiple biologics with potential relevance for treatment stratification, use of validated response criteria, and high data completeness and quality.

## 4. Materials and Methods

Patients with psoriasis treated with biologics at the Department of Dermatology and Allergy, Herlev and Gentofte Hospital, Denmark, were invited to participate. Before inclusion, all patients gave informed consent (Ethics Committee of the Capital Region of Denmark, H-19036920). Clinical data and response to treatment were registered and collected from patient records. Blood samples were collected during routine visits, and single-nucleotide polymorphism (SNP) analysis was performed at the Institute for Inflammation Research, Rigshospitalet, Copenhagen. Patients receiving any type of biologics were eligible if the Psoriasis Area and Severity Index (PASI) after three months (±45 days) was available. Response was defined as absolute PASI ≤ 2 after three months of treatment, as this is the preferred response criterion in the real world [6,31,32]. The percentage of patients responding was calculated for patients treated with TNF inhibitors (TNFi, adalimumab, etanercept, and infliximab), IL-12/23 inhibitors (IL-12/23i, ustekinumab), and IL-17 cytokine or receptor inhibitors (IL-17i/IL-17Ri, brodalumab, ixekizumab, and secukinumab) according to class.

### 4.1. Genetic Analyses

SNPs outside the HLA region were chosen based on their previous reported association with response to biologics in psoriasis or other chronic immune-mediated diseases [12]. In total, 67 SNPs were chosen (Supplementary Table S1). SNPs were genotyped using an in-house multiplexed bead-based assay described in detail elsewhere [33]. In brief, allele-specific primers were labeled in an allele-specific primer extension (ASPE)–reaction on polymerase chain reaction (PCR)-amplified SNP sites. The labeled ASPE primers were subsequently hybridized to MagPlex-TAG beads (Luminex Corporation, Austin, TX, USA) for detection and counting on the Luminex platform (Luminex Corporation, Austin, TX, USA). As an internal quality control, we included an in-house test for sex determination based on a six base-pair difference between the X-Y homologous amelogenin genes [34]. Genotyping for sex was in accordance with the demographic data. Additionally, eight DNA samples with known genotypes were obtained from the Coriell Cell Repository (Camden, NJ, USA) and were included in all plate runs as controls, together with no-template PCR-negative controls. To further verify the obtained genotypes, approximately 5% of the samples were randomly selected for retyping in a separate analysis. All re-typings were consistent with the first typing. Additional methodological information is available from the corresponding author upon request.

### 4.2. Statistical Analyses

Odds ratios (OR) with 95% confidence intervals (CI) were calculated with logistic regression assessing association between genetic variants and response to biologics. Response was defined as achievement of an absolute PASI ≤ 2 after three months (±45 days). For genetic exposure, we used an additive model with the minor alleles as exposure. We present OR crude and in an adjusted model adjusting for sex, age, psoriatic arthritis, and biologic nativity. Due to all genetic variants previously being associated with response to biologics, a *p*-value < 0.05 without correction for multiple testing was considered significant. All data management and statistical analyses were conducted using R Version 4.3.0.

## 5. Conclusions

The study replicates several findings from previous studies in treatment with TNFi and suggests genetic variants previously associated with response to biologics for inflammatory diseases are also associated with response to IL-17i/IL-17Ri and IL-12/23i in patients with psoriasis. Two genetic variants, IRAK-3 (*rs11541076*) and CD84 (*rs6427528*), showed potential for use in treatment stratification. However, these findings should be interpreted with caution, and further studies in diverse cohorts will be necessary to confirm the robustness and generalizability of these associations.

## Figures and Tables

**Table 1:** Clinical characteristics of patients.

	TNFi N = 319	IL-12/23i N = 109	IL-17i N = 146
Age, years, mean (SD)	43.9 (15.4)	40.7 (14.9)	46.4 (15.3)
Sex, male, *n* (%)	211 (66.1%)	78 (71.6%)	95 (65.2%)
BMI, mean (SD)	29.3 (6.8)	28.8 (6.3)	29.7 (8.5)
Baseline PASI, mean (SD)	10.2 (6.2)	10.0 (5.7)	9.4 (5.1)
PsA, *n* (%)	73 (22.9%)	17 (15.7%)	34 (23.2%)
Biologic naïve	228 (71.5%)	59 (53.9%)	39 (26.7%)

Abbreviations: TNFi, tumor necrosis factor α inhibitors; IL-12/23i, interleukin 12/23 inhibitor; IL-17i, interleukin 17 inhibitors; *n*, number, PASI, psoriasis area and severity index, PsA, psoriatic arthritis.

**Table 2:** Any significant association between genetic variants and the achievement of PASI ≤ 2 after 3 months across all drug classes. The table presents crude odds ratios (OR) and OR adjusted for age, sex, biologic naivety, and psoriatic arthritis.

SNP (rs Number Major/Minor Allele)	TNFi (*n* = 319) ORcrude (95% CI), *p*-Value	TNFi (*n* = 319) ORadj (95% CI), *p*-Value	IL-12/23i (*n* = 109) ORcrude (95% CI), *p*-Value	IL-12/23i (*n* = 109) ORadj (95% CI), *p*-Value	IL-17i/IL-17Ri (*n* = 146) ORcrude (95% CI), *p*-Value	IL-17i/IL-17Ri (*n* = 146) ORadj (95% CI), *p*-Value
PSTPIP1 (*rs2254441 G/A*) MAF = 0.21	1.67 (1.13–2.47), **0.0096**	1.70 (1.14–2.53), **0.0081**	1.01 (0.56–1.82), 0.96	0.92 (0.50–1.71), 0.80	1.08 (0.60–1.94), 0.79	0.94 (0.50–1.75), 0.85
TNFRSF1A (*rs4149570 C/A*) MAF = 0.38	0.68 (0.48–0.96), **0.032**	0.66 (0.46–0.94), **0.023**	0.74 (0.43–1.28), 0.29	0.74 (0.42–1.31), 0.30	0.89 (0.53–1.50), 0.67	0.92 (0.53–1.61), 0.78
CASP9 (*rs4645983 G/A*) MAF = 0.23	1.10 (0.69–1.76), 0.66 (*n* = 224)	1.11 (0.69–1.79), 0.64 (*n* = 224)	0.78 (0.39–1.56), 0.49 (*n* = 78)	0.68 (0.32–1.43), 0.31 (*n* = 78)	2.48 (1.09–5.68), **0.030** (*n* = 101)	2.21 (0.90–5.40), 0.081 (*n* = 101)
TLR5 (*rs5744174 A/G*) MAF = 0.45	0.92 (0.66–1.28), 0.63	0.92 (0.66–1.28), 0.63	1.08 (0.64–1.77), 0.77	0.91 (0.53–1.56), 0.74	2.00 (1.14–3.50), **0.015**	1.99 (1.095–3.64), **0.024**
CD84 (*rs6427528 G/A*) MAF = 0.099	0.53 (0.30–0.91), **0.023**	0.53 (0.30–0.92), **0.026**	1.64 (0.68–3.93), 0.26	1.53 (0.60–3.90), 0.36	0.50 (0.25–0.98), **0.045**	0.51 (0.25–1.05), 0.066
GFRA1 (*rs7070180 C/T*) MAF = 0.25	1.21 (0.84–1.75), 0.28 (*n* = 315)	1.23 (0.85–1.78), 0.26 (*n* = 315)	2.14 (1.10–4.14), **0.024** (*n* = 108)	2.17 (1.057–4.48), **0.034** (*n* = 108)	1.10 (0.58–2.09), 0.76 (*n* = 144)	1.08 (0.54–2.13), 0.82 (*n* = 144)
STAT4 (*rs7574865 G/T*) MAF = 0.19	0.58 (0.38–0.90), **0.014** (*n* = 264)	0.60 (0.39–0.92), **0.020** (*n* = 264)	0.89 (0.42–1.86), 0.76 (*n* = 83)	0.72 (0.31–1.63), 0.43 (*n* = 83)	1.12 (0.62–2.05), 0.69 (*n* = 127)	1.27 (0.66–2.43), 0.45 (*n* = 127)
CD226 (*rs763361 C/T*) MAF = 0.48	0.78 (0.58–1.06), 0.11	0.-79 (0.58–1.07), 0.13	1.00 (0.59–1.67), 0.10	0.94 (0.54–1.63), 0.83	1.85 (1.14–3.02), **0.012**	1.80 (1.090–2.99), **0.021**
LINC02964 (*rs921720 G/A*) MAF = 0.39	0.90 (0.64–1.27), 0.56 (*n* = 317)	0.92 (0.65–1.29), 0.63 (*n* = 317)	0.90 (0.50–1.62), 0.73 (*n* = 108)	0.87 (0.45–1.66), 0.67 (*n* = 108)	0.53 (0.30–0.92), **0.025** (*n* = 144)	0.45 (0.25–0.83), **0.0099** (*n* = 144)
IL-18 (*rs187238 C/G*) MAF = 0.27	1.20 (0.80–1.80), 0.36 (*n* = 264)	1.17 (0.78–1.78), 0.43 (*n* = 264)	2.03 (0.99–4.14), 0.051 (*n* = 87)	2.59 (1.11–6.02), **0.026** (*n* = 87)	1.20 (0.65–2.18), 0.55 (*n* = 129)	1.22 (0.65–2.30), 0.53 (*n* = 129)
IRAK3 (*rs11541076 A/T*) MAF = 0.15	2.56 (1.22–5.37), **0.012** (*n* = 127)	2.47 (1.13–5.38), **0.022** (*n* = 127)	0.38 (0.084–1.72), 0.21 (*n* = 44)	0.23 (0.044–1.28), 0.094 (*n* = 44)	2.55 (0.70–9.22), 0.15 (*n* = 56)	2.21 (0.57–8.55), 0.24 (*n* = 56)
TNF (*rs1799724 C/T*) MAF = 0.10	0.86 (0.51–1.45), 0.57	0.84 (0.50–1.43), 0.54	0.61 (0.24–1.53), 0.29	0.52 (0.19–1.38), 0.19	0.47 (0.22–0.99), **0.049**	0.61 (0.27 (0.27–1.37), 0.23

Abbreviations: TNFi, tumor necrosis factor α inhibitors; IL-12/23i, interleukin 12/23 inhibitor; IL-17i/IL-17Ri, interleukin 17 inhibitors/interleukin 17 receptor inhibitors; *n*, number; PASI, psoriasis area and severity index; MAF, minor allele frequency. Certain SNPs did not have full calls, and the number of patients on which the model is based is reported for these. *p*-value < 0.05 are marked in bold.

## Data Availability

The data that support the findings of this study are available from the corresponding author upon reasonable request.

## Supplementary Materials

The following supporting information can be downloaded at: https://www.mdpi.com/article/10.3390/ijms26188998/s1.
