# Supplementary material for: The Association Between Genetics and Response to Treatment with Biologics in Patients with Psoriasis
Source: Int J Mol Sci. 2025 Sep 16;26(18):8998. doi: 10.3390/ijms26188998 (PMC12469637; doi:10.3390/ijms26188998)
Supplement: Supplementary file 1 [file ijms-26-08998-s001.zip › ijms-3816873-supplementary.pdf]

Supplementary Table S1. Association between genetic variants and PASI 2 after 3 months.

SNP (major/minor allele)	TNFi (n=319) OR_{unadj} (95% CI), p-value	TNFi (n=319) OR_{adj} (95% CI), p-value	Ustekinumab (n=109) OR_{unadj} (95% CI), p-value	Ustekinumab (n=109) OR_{adj} (95% CI), p-value	IL-17i (n=146) OR_{unadj} (95% CI), p-value	IL-17i (n=146) OR_{adj} (95% CI), p-value
rs1061622 T/G MAF=0.24	1.11 (0.77-1.58), 0.56	1.095 (0.76-1.56), 0.61	0.69 (0.37-1.30), 0.25	0.60 (0.30-1.17), 0.13	1.12 (0.60-2.082), 0.71	1.084 (0.55-2.11), 0.81
rs10754558 C/G MAF=0.39	0.81 (0.58-1.15), 0.25	0.82 (0.58-1.16), 0.26	0.88 (0.49-1.56), 0.67	0.93 (0.52-1.69), 0.83	1.14 (0.66-1.94), 0.63	1.31 (0.75-2.31), 0.34
rs1077773 A/G MAF=0.46	0.94 (0.68-1.31), 0.72	0.93 (0.66-1.29), 0.66	1.11 (0.63-1.96), 0.70	1.016 (0.55-1.85), 0.95	1.14 (0.70-1.87), 0.58	1.22 (0.71-2.092), 0.46
rs10865035 G/A MAF=0.45	1.045 (0.75-1.44), 0.79 (n=318)	1.031 (0.74-1.43), 0.85 (n=318)	0.86 (0.50-1.49), 0.60	0.87 (0.48-1.57), 0.65	0.86 (0.53-1.39), 0.55 (n=145)	0.97 (0.58-1.63), 0.92 (n=145)
rs10919563 G/A MAF=0.14	1.32 (0.88-1.98), 0.16	1.36 (0.90-2.048), 0.13	1.48 (0.63-3.48), 0.36	1.33 (0.54-3.27), 0.53	0.98 (0.49-1.92), 0.95	1.091 (0.54-2.20), 0.80
rs10925026 A/C MAF=0.37	1.096 (0.79-1.51), 0.57 (n=318)	1.10 (0.79-1.52), 0.56 (n=318)	1.84 (0.99-3.42), 0.052	1.69 (0.87-3.28), 0.11	1.16 (0.65-2.067), 0.60 (n=145)	1.25 (0.68-2.32), 0.46 (n=145)
rs11126740 G/A MAF=0.32	0.93 (0.66-1.29), 0.66	0.92 (0.66-1.28), 0.63	0.81 (0.45-1.45), 0.48	0.66 (0.35-1.25), 0.20	1.19 (0.71-2.011), 0.50	1.083 (0.63-1.86), 0.77
rs11209026 G/A MAF=0.043	1.11 (0.55-2.32), 0.75 (n=318)	1.10 (0.55-2.21), 0.78 (n=318)	0.42 (0.10-1.69), 0.22	0.52 (0.12-2.28), 0.39	0.80 (0.22-2.91), 0.74 (n=144)	1.051 (0.27-4.052), 0.94 (n=144)
rs1143623 C/G MAF=0.26	1.092 (0.76-1.55), 0.62	1.077 (0.75-1.53), 0.68	1.008 (0.51-1.96), 0.98	1.060 (0.52-2.13), 0.87	0.81 (0.46-1.41), 0.46	0.89 (0.50-1.59), 0.71
rs1143627 A/G MAF=0.31	1.80 (0.69-4.67), 0.22 (n=46)	3.41 (0.95-12.21), 0.058 (n=46)	NA	NA	0.43 (0.12-1.44), 0.17 (n=24)	0.69 (0.11-4.23), 0.68 (n=24)
rs1143634 G/A MAF=0.26	0.78 (0.49-1.26), 0.32 (n=216)	0.76 (0.47-1.23), 0.27 (n=216)	1.44 (0.69-3.003), 0.33 (n=79)	1.59 (0.73-3.46), 0.23 (n=79)	0.61 (0.32-1.16), 0.13 (n=102)	0.73 (0.37-1.43), 0.36 (n=102)
rs11541076 A/T MAF=0.15	2.56 (1.22-5.37), **0.012** (n=127)	2.47 (1.13-5.38), **0.022** (n=127)	0.38 (0.084-1.72), 0.21 (n=44)	0.23 (0.044-1.28), 0.094 (n=44)	2.55 (0.70-9.22), 0.15 (n=56)	2.21 (0.57-8.55), 0.24 (n=56)
rs11652075 C/T MAF=0.44	1.086 (0.79-1.48), 0.60	1.10 (0.80-1.51), 0.54	1.070 (0.60-1.89), 0.81	1.14 (0.62-2.094), 0.67	0.84 (0.51-1.36), 0.47	0.89 (0.54-1.49), 0.68
rs11938228 C/A MAF=0.30	1.15 (0.80-1.63), 0.43	1.13 (0.78-1.58), 0.55	1.23 (0.71-2.14), 0.44	1.20 (0.67-2.15), 0.53	0.94 (0.52-1.69), 0.84	0.92 (0.49-1.73), 0.81
rs13190932 G/A MAF=0.066	1.32 (0.77-2.25), 0.30	1.27 (0.74-2.19), 0.38	1.46 (0.51-4.12), 0.47	1.29 (0.44-3.78), 0.64	0.87 (0.34-2.24), 0.78	0.80 (0.29-2.15), 0.66
rs1799724 C/T MAF=0.10	0.86 (0.51-1.45), 0.57	0.84 (0.50-1.43), 0.54	0.61 (0.24-1.53), 0.29	0.52 (0.19-1.38), 0.19	0.47 (0.22-0.99), **0.049**	0.61 (0.27 (0.27-1.37), 0.23
rs1800629 G/A MAF=0.18	1.40 (0.34-5.76), 0.63 (n=31)	0.79 (0.10-5.79), 0.81 (n=31)	NA	NA	NA	NA
rs1800795 G/C MAF=0.42	1.14 (0.82-1.58), 0.41	1.15 (0.83-1.60), 0.38	1.22 (0.68-2.18), 0.49	1.32 (0.71-2.44), 0.37	1.65 (0.94-2.88), 0.077	1.54 (0.85-2.79), 0.15
rs1801274 A/G MAF=0.49	1.19 (0.88-1.62), 0.24 (n=318)	1.17 (0.86-1.59), 0.30 (n=318)	1.028 (0.59-1.78), 0.92	1.096 (0.59-2.034), 0.77	0.65 (0.40-1.069), 0.090 (n=145)	0.60 (0.35-1.013), 0.056 (n=145)
rs187084 A/G MAF=0.41	1.20 (0.88-1.65), 0.23	1.18 (0.86-1.62), 0.28	0.98 (0.56-1.71), 0.95	1.027 (0.57-1.83), 0.92	0.88 (0.54-1.42), 0.61	0.81 (0.48-1.36), 0.44

rs187238 C/G MAF=0.27	1.20 (0.80-1.80), 0.36 (n=264)	1.17 (0.78-1.78), 0.43 (n=264)	2.029 (0.99-4.14), 0.051 (n=87)	2.59 (1.11-6.02), **0.026** (n=87)	1.20 (0.65-2.18), 0.55 (n=129)	1.22 (0.65-2.30), 0.53 (n=129)
rs191190 T/C MAF=0.37	1.13 (0.81-1.57), 0.44	1.13 (0.81-1.57), 0.45	1.21 (0.70-2.077), 0.48	1.40 (0.78-2.49), 0.25	1.24 (0.71-2.18), 0.44	1.27 (0.70-2.30), 0.41
rs1946518 G/T MAF=0.40	1.016 (0.73-1.40), 0.92	0.98 (0.71-1.36), 0.93	1.39 (0.82-2.34), 0.21	1.40 (0.80-2.46), 0.23	1.32 (0.80-2.17), 0.27	1.25 (0.73-2.11), 0.40
rs2066808 A/G MAF=0.046	1.37 (0.67-2.82), 0.38	1.40 (0.67-2.93), 0.35	1.023 (0.29-3.57), 0.97	1.016 (0.27-3.71), 0.98	0.63 (0.19-2.13), 0.46	0.70 (0.19-2.54), 0.59
rs2145623 G/C MAF=0.34	1.17 (0.83-1.64), 0.35	1.19 (0.84-1.68), 0.30	1.002 (0.57-1.76), 0.99	0.94 (0.52-1.69), 0.84	0.92 (0.55-1.54), 0.76	0.90 (0.52-1.55), 0.71
rs2206593 G/A MAF=0.053	1.19 (0.59-2.39), 0.62	1.18 (0.58-2.43), 0.63	0.55 (0.17-1.78), 0.32	0.55 (0.17-1.80), 0.32	0.37 (0.13-1.034), 0.058	0.37 (0.12-1.12), 0.079
rs2243188 C/A MAF=0.25	1.23 (0.87-1.72), 0.22	1.22 (0.87-1.71), 0.24	1.21 (0.66-2.21), 0.52	1.10 (0.58-2.089), 0.76	1.28 (0.75-2.18), 0.36	1.10 (0.63-1.94), 0.72
rs2254441 G/A MAF=0.21	1.67 (1.13-2.47), **0.0096**	1.70 (1.14-2.53), **0.0081**	1.012 (0.56-1.82), 0.96	0.92 (0.50-1.71), 0.80	1.082 (0.60-1.94), 0.79	0.94 (0.50-1.75), 0.85
rs2430561 T/A MAF=0.45	0.91 (0.66-1.26), 0.60	0.91 (0.66-1.26), 0.58	0.99 (0.58-1.72), 0.99	1.045 (0.58-1.85), 0.88	0.68 (0.41-1.11), 0.12	0.69 (0.41-1.16), 0.16
rs27524 G/A MAF=0.38	0.86 (0.62-1.18), 0.37	0.86 (0.62-1.18), 0.36	1.61 (0.93-2.78), 0.085	1.59 (0.87-2.92), 0.13	0.99 (0.60-1.65), 0.99	1.056 (0.62-1.78), 0.84
rs2916205 T/C MAF=0.11	1.22 (0.74-2.005), 0.43	1.19 (0.72-1.97), 0.49	0.59 (0.24-1.43), 0.24	0.42 (0.16-1.095), 0.076	1.13 (0.54-2.37), 0.72	1.27 (0.72-1.97), 0.49
rs3213094 C/T MAF=0.13	0.79 (0.50-1.25), 0.32	0.78 (0.49-1.25), 0.30	1.37 (0.63-2.96), 0.41	1.086 (0.46-2.53), 0.84	1.10 (0.60-2.020), 0.74	0.94 (0.49-1.80), 0.85
rs3217713 T/A MAF=0.24	0.92 (0.64-1.33), 0.68	0.94 (0.65-1.36), 0.76	1.41 (0.77-2.59), 0.25	1.49 (0.79-2.83), 0.21	0.92 (0.50-1.71), 0.81	1.059 (0.54-2.050), 0.86
rs361525 G/A MAF=0.13	1.31 (0.81-2.11), 0.26	1.28 (0.79-2.087), 0.30	1.35 (0.59-3.071), 0.46	1.23 (0.52-2.89), 0.63	1.45 (0.64-3.32), 0.36	1.086 (0.45-2.61), 0.85
rs397211 T/C MAF=0.26	0.88 (0.61-1.28), 0.52	0.89 (0.61-1.30), 0.57	0.93 (0.52-1.66), 0.82	0.97 (0.63-1.77), 0.92	1.025 (0.59-1.75), 0.92	1.13 (0.63-2.029), 0.66
rs4149570 C/A MAF=0.38	0.68 (0.48-0.96), **0.032**	0.66 (0.46-0.94), **0.023**	0.74 (0.43-1.28), 0.29	0.74 (0.42-1.31), 0.30	0.89 (0.53-1.50), 0.67	0.92 (0.53-1.61), 0.78
rs4645983 G/A MAF=0.23	1.10 (0.69-1.76), 0.66 (n=224)	1.11 (0.69-1.79), 0.64 (n=224)	0.78 (0.39-1.56), 0.49 (n=78)	0.68 (0.32-1.43), 0.31 (n=78)	2.48 (1.087-5.68), **0.030** (n=101)	2.21 (0.90-5.40), 0.081 (n=101)
rs4819554 A/G MAF=0.17	0.88 (0.57-1.36), 0.59	0.87 (0.57-1.35), 0.55	0.96 (0.48-1.89), 0.90	0.98 (0.48-1.98), 0.96	1.050 (0.53-2.060), 0.88	0.99 (0.49-2.002), 0.98
rs5030728 G/A MAF=0.31	0.97 (0.69-1.36), 0.86	0.98 (0.70-1.39), 0.95	1-19 (0.67-2.097), 0.54	1.33 (0.73-2.44), 0.34	0.88 (0.51-1.51), 0.64	0.91 (0.52-1.60), 0.76
rs5744174 A/G MAF=0.45	0.92 (0.66-1.28), 0.63	0.92 (0.66-1.28), 0.63	1.077 (0.64-1.77), 0.77	0.91 (0.53-1.56), 0.74	2.00 (1.14-3.50), **0.015**	1.99 (1.095-3.64), **0.024**
rs6427528 G/A MAF=0.099	0.53 (0.30-0.91), **0.023**	0.53 (0.30-0.92), **0.026**	1.64 (0.68-3.93), 0.26	1.53 (0.60-3.90), 0.36	0.50 (0.25-0.98), **0.045**	0.51 (0.25-1.047), 0.066
rs6661932 T/C MAF=0.49	1.095 (0.79-1.50), 0.57	1.15 (0.83-1.60), 0.38	1.58 (0.92-2.71), 0.094	1.45 (0.82-2.57), 0.19	1.57 (0.99-2.49), 0.051	1.46 (0.89-2.39), 0.12
rs6908425 C/T MAF=0.17	1.006 (0.66-1.53), 0.97	0.99 (0.64-1.51), 0.96	1.29 (0.61-2.72), 0.49	1.30 (0.59-2.84), 0.50	1.20 (0.62-2.35), 0.57	1.096 (0.55-2.17), 0.79

rs7070180 C/T MAF=0.25	1.21 (0.84-1.75), 0.28 (n=315)	1.23 (0.85-1.78), 0.26 (n=315)	2.14 (1.10-4.14), **0.024** (n=108)	2.17 (1.057-4.48), **0.034** (n=108)	1.10 (0.58-2.092), 0.76 (n=144)	1.079 (0.54-2.13), 0.82 (n=144)
rs7574865 G/T MAF=0.19	0.58 (0.38-0.90), **0.014** (n=264)	0.60 (0.39-0.92), **0.020** (n=264)	0.89 (0.42-1.86), 0.76 (n=83)	0.72 (0.31-1.63), 0.43 (n=83)	1.12 (0.62-2.046), 0.69 (n=127)	1.27 (0.66-2.43), 0.45 (n=127)
rs763110 C/T MAF=0.34	0.76 (0.54-1.060), 0.10	0.75 (0.54-1.056), 0.10	0.84 (0.48-1.49), 0.56	0.80 (0.43-1.46), 0.47	0.77 (0.46-1.28), 0.32	0.71 (0.41-1.23), 0.22
rs763361 C/T MAF=0.48	0.78 (0.58-1.062), 0.11	0.-79 (0.58-1.072), 0.13	1.00 (0.59-1.67), 0.100	0.94 (0.54-1.63), 0.83	1.85 (1.14-3.020), **0.012**	1.80 (1.090-2.99), **0.021**
rs763780 T/C MAF=0.062	1.53 (0.79-2.98), 0.20	1.46 (0.74-2.86), 0.26	1.00 (0.37-2.65), 0.99	1.12 (0.39-3.18), 0.82	0.87 (0.34-2.22), 0.77	0.89 (0.32-2.42), 0.82
rs767455 T/C MAF=0.42	1.12 (0.80-1.57), 0.50 (n=318)	1.14 (0.81-1.60), 0.45 (n=318)	0.88 (0.52-1.50), 0.65 (n=108)	0.87 (0.50-1.50), 0.62 (n=108)	0.82 (0.48-1.38), 0.46	0.78 (0.45-1.36), 0.38
rs774359 T/C	1.10 (0.75-1.61), 0.60 MAF=0.25	1.10 (0.75-1.61), 0.61 MAF=0.25	1.34 (0.75-2.40), 0.31 MAF=0.25	1.40 (0.76-2.60), 0.27 MAF=0.25	1.27 (0.64-2.52), 0.48 MAF=0.25	1.44 (0.69-3.015), 0.32 MAF=0.25
rs8177374 C/T MAF=0.13	1.091 (0.55-2.14), 0.80 (n=175)	1.21 (0.60-2.45), 0.58 (n=175)	1.35 (0.50-3.68), 0.54 (n=58)	1.29 (0.45-3.70), 0.63 (n=58)	0.42 (0.15-1.18), 0.10 (n=80)	0.36 (0.12-1.11), 0.077 (n=80)
rs921720 G/A MAF=0.39	0.90 (0.64-1.27), 0.56 (n=317)	0.92 (0.65-1.29), 0.63 (n=317)	0.90 (0.50-1.62), 0.73 (n=108)	0.87 (0.45-1.66), 0.67 (n=108)	0.53 (0.30-0.92), **0.025** (n=144)	0.45 (0.25-0.83), **0.0099** (n=144)

Abbreviations: TNFi, tumor necrosis factor α inhibitors; IL-12/23i, interleukin 12/23 inhibitor; IL-17i, interleukin 17 inhibitors; n, number, PASI, psoriasis area and severity index; MAF, minor allele frequency.
Adjusted analyses were adjusted for age, sex, biologic naivety, and psoriatic arthritis.
